# Sialic Acid-Siglec Axis as Molecular Checkpoints Targeting of Immune System: Smart Players in Pathology and Conventional Therapy

**DOI:** 10.3390/ijms21124361

**Published:** 2020-06-19

**Authors:** Przemyslaw Wielgat, Karol Rogowski, Katarzyna Niemirowicz-Laskowska, Halina Car

**Affiliations:** 1 Department of Clinical Pharmacology, Medical University of Bialystok, Waszyngtona 15A, 15-274 Bialystok, Poland; hcar@umb.edu.pl; 2 Department of Experimental Pharmacology, Medical University of Bialystok, Szpitalna 37, 15-295 Bialystok, Poland; karolrogovsky@gmail.com (K.R.); katia146@wp.pl (K.N.-L.)

**Keywords:** sialic acid, Siglec, checkpoint axis, immune system, pathology, conventional therapy

## Abstract

The sialic acid-based molecular mimicry in pathogens and malignant cells is a regulatory mechanism that leads to cross-reactivity with host antigens resulting in suppression and tolerance in the immune system. The interplay between sialoglycans and immunoregulatory Siglec receptors promotes foreign antigens hiding and immunosurveillance impairment. Therefore, molecular targeting of immune checkpoints, including sialic acid-Siglec axis, is a promising new field of inflammatory disorders and cancer therapy. However, the conventional drugs used in regular management can interfere with glycome machinery and exert a divergent effect on immune controlling systems. Here, we focus on the known effects of standard therapies on the sialoglycan-Siglec checkpoint and their importance in diagnosis, prediction, and clinical outcomes.

## 1. Introduction

The immune homeostasis is a complex and precise mechanism that underlies tissue environment control, regeneration, and repair processes, as well as the surveillance of pathogens and malignancies [1,2]. All events controlled by the immune system depend on the cellular interactions that maintain the balance between tolerance and defense processes. The communication between host cells and their environment recruits the cellular and molecular mechanisms responsible for the recognition, adhesion, and secretory activity [3]. Recent advances in immunology show that targeting the molecules underlying immune homeostasis is a promising therapeutic tool for inflammation, autoimmunity, cancer, and neurodegeneration [4,5]. The immune checkpoints are the system of regulatory proteins that play a critical role in self-tolerance processes and prevent autoimmune reactions against self-produced antigens [6]. The interplay between stimulatory or inhibitory checkpoint molecules with their specific ligands modulates cellular functions to avoid immune injury. However, the mechanisms underlying these processes are not fully understood. Moreover, the molecular mimicry of checkpoint systems by pathogens leads to cross-reactivity with host antigens resulting in suppression and tolerance in the immune system [7,8,9]. The clinical data support that the prolonged exposure to bacterial and viral antigens leads to overexpression of several checkpoint receptors, e.g., programmed cell death protein 1 (PD-1) and cytotoxic T-lymphocyte-associated protein 4 (CTLA-4) in effector lymphocytes that provide negative signals and induce reversible exhaustion state. The anti-PD-1 antibody-based directed therapies have been shown to have a beneficial effect on malignant cell clearance [10,11,12,13]. The PD-L1 and CTLA-4 targeting have been introduced successfully into oncological practice, and the combination immunotherapy and multiple immunomodulatory targets open promising therapeutic strategies [14].

A growing body of evidence supports the role of sialoglycans at various clinical stages of immune-based pathologies [15,16]. Since sialylated glycans are involved in many biological processes, their frequently altered expression, as well as recognition by individual sialic acid-binding immunoglobulin-like lectins (Siglecs), can be related to the increased progression of the pathological processes [17,18,19]. This review briefly focuses on the engagement of the sialic acid-Siglec axis in some pathophysiological processes and its importance in routine clinical practice.

## 2. Sialic Acid and Immune Recognition

More than 50 nine-carbon monosugars derived from neuraminic acid belong to the family of sialic acids, among which N-acety1-5-neuraminic acid also called sialic acid (SA, Neu5Ac, NANA) is the most common form found in cell membrane glycoproteins and body fluids [20]. The sialic acid is ubiquitously expressed, typically at the terminal position of glycoproteins and lipids in the glycosylation process, resulting in co-translational and posttranslational modifications of approximately 80% of cell proteins [21]. Sialylation as the final stage of glycosylation is based on the balance achieved by the expression and activity of sialyltransferases and sialidases involved in the decoration of sugar chains, and on the sialic acid precursors contained in nutrient resources, as well as the expression of several metabolic enzymes implicated in the synthesis and conversion of sialic acid molecules. The attachment of sialic acid enhances the complexity of the glycosylation processes and results in wide microheterogeneity of glycoconjugates, which can be used to predict the occurrence of pathology, diagnosis, and therapy monitoring [22,23]. In contrast to the stable and reproducible glycosylation pattern under normal conditions, the unbalanced sialylation processing enzymes lead to the dramatic differences in sialic acid expression. It is of particular importance in the context of immune recognition processes underlying chronic inflammatory diseases and immune tolerance in cancer [24,25,26,27]. The biological recognition processes are closely linked to the biological function of sialic acids and include the regulation of adhesion that occurs from cell-cell and cell-extracellular matrix (ECM) interaction [28,29]. Binding of specific membrane sialoglycoproteins is the first step in the adsorption of pathogens on host cell membranes and further colonization of tissues and organs. This process has been confirmed in bacterial (*Escherichia coli*, *Streptococcus suis*), viral (influenza, *Cardiovirus*, *Paramyxovirus*), and protozoan (*Plasmodium falciparum*) infections [30,31,32,33,34,35]. Sialoglycans, especially sialo-Lewis^a,b,x,y^ epitopes, play a crucial role in the interaction with selectins, which are the molecular basis of adhesion processes linked to the migration of immune cells to the target organs through the vascular endothelium and outside the circulatory system. Thus, the sialic acid-mediated negative charge on membranes reduces the mutual adhesiveness of cells, which underlies the migration of highly sialylated cancer cells in the metastatic process [36]. In addition, the aberrant sialic acids mask the underlying glycan structure, thereby avoiding recognition by other lectins such as galectins and C-type lectins [37]. The host’s immune system, whose cells express sialo-Lewis antigens does not produce specific antibodies and allows the invasion of sialo-Lewis positive pathogens by way of molecular mimicry. Many malignancies use this mechanism to hide their epitopes, which inhibits the complement activation pathway to reduce immunogenicity, recruits of plasma factor H to control of alternative complement pathways. Furthermore, the sialic acid epitopes protect the human colon mucins from the clearance by liver receptors, including the hepatocyte asialoglycoprotein receptor (ASGPR), macrophage galactose lectin-1,-2 (MGL-1,-2), hyaluronic acid receptor for endocytosis, and scavenger receptors (SRs) [17,38,39]. The digestion with neuraminidase becomes cells more immunogenic, and the weaker antigenic sites more accessible. Loss of membrane sialic acid in lymphoid cells increases their migration to the liver and makes them more deformable and phagocytic [40]. Recent advances in glycoimmunology indicate the interplay between the cell membrane sialylated glycans with Siglec immune receptors as a new checkpoint axis in the regulation of the immune system [41,42]. The human CD33-related Siglecs, as well as their mouse homologs, form a major subfamily of the Siglecs characterized by the specificity of distribution in the immune cells and recognition of sugar products [43,44]. Differences in the structure of the intracellular domain of Siglecs determine the activating or suppressive signaling pathways responsible for the function of the immune cells. Posttranslational glycosylation of cell adhesion molecules (CAMs) plays a pivotal role in regulating cell proliferation, differentiation, migration, and survival that underlie ontogenetic development and cellular plasticity [45]. In the central nervous system (CNS), the glycan-dependent cross-talk between neurons, glia, and microglia form a balance between *synapse* formation, potentiation, and removal, thereby maintaining homeostasis of the brain by controlling the tissue architecture, microenvironment, and defense reactions [46,47]. Clinical observations, animal models, and in vitro co-culture systems confirmed the significance of glycoconjugates sialylation in innate immunity and its relationship with development, cognition, regeneration, and aging [17,48]. In the brain, sialylated glycocalyx is recognized by Siglec-expressing microglial cells that normalize normal brain wiring, as well as various type leukocytes infiltrating the infected and/or damaged structures [49,50]. The polysialylated derivatives of neural CAMs (PSA-NCAMs) are known as a specific ligand for the microglial Siglec-11 receptor, which transduces an immunosuppressive signal and inhibits several immune functions. The binding of PSA with the Siglec-11 receptor in the neuron-microglia co-culture system was closely associated with limited immune function. It may reflect the control mechanism, called cis-interaction, which prevents the autoimmune processes in the healthy CNS [50]. The imbalance between sialidases and sialyltransferases activities, as a result of pathology or exposure to degenerative factors, disturbs the sialylation pattern, and modulates the function of “On” and “Off” signaling system. Interestingly, the enzymatic removal of sialic acid reduces the neuritic density and the number of perikaryons and induces changes in the morphology of microglia expressed by the transformation of the resting to the activated form [50]. In line with this observation, selective enzymatic removal of sialic acids attached by α2,3 and α2,6 linkages reduces the reactivity of the suppressive Siglec-F receptor protein to its ligands on the neuronal surface, which can be part of the mechanism of neuronal protection and homeostasis in the brain [50].

## 3. Sialic Acid-Siglec Checkpoint in Human Pathology

The molecular pattern of glycosylation has an essential role in biological recognition and could predict the involvement of the immune system in pathology initiation and progression. Recent advances in glycobiology are focused on the prognostic value of sialylated epitopes as markers of pathology [44,51]. The applied experimental models and analyzed clinical material referring to the various human pathologies demonstrated changes in the level of cell membrane sialoglycans. Sialylation processes pretend to be a useful prognostic marker and the potential target for drug development as well as an indicator in monitored therapy [17]. To date, serum total sialic acid as well as lipid- and protein-bound sialic acids are the fields of clinical interest in their importance as diagnostic markers of pathology. The assessment of differences in the level of free sialic acid and patterns of sialylation of particular glycosaminoglycans is characterized by the various methodological approaches in current glycoscience. The quantification of serum and plasma sialic acids by colorimetric, fluorimetric, and enzymatic methods confirmed their significance as a prognostic factor in clinical practice. However, multiple interferences from substances present in biological samples are the strong limitation for routine use of these analyses [52]. Since the immunological methods have been developed, the specific monoclonal antibodies and labeled sialic acid-binding lectins are widely used in the evaluation of basement membrane sialic acid composition by electrophoretic and ELISA methods in the studies of cancer biology [53,54]. The latest advances in this field are enriched by the development of mass spectrometry (MS) with high resolution and mass accuracy that allows analyzing glycans in terms of structure [55,56]. For example, the recent analysis of the sialic acid linkages of the glycome of the epithelial ovarian cancer (EOS) patients by the matrix-assisted laser desorption/ionization time-of-flight (MALDI-TOF) mass spectrometry revealed significant differences in α2.3-linked/α2.6-linked sialic acid ratio in EOS patients when compared to healthy individuals [57].

Most Siglecs participate in negative signal transduction resulting in the downregulation of the immune response and are critical for self-tolerance processes and prevent autoimmune reactions against self-produced antigens [43]. Sialylated glycoconjugates belong to the self-associated molecular patterns (SAMPs) that bind to the individual immunoreceptor tyrosine-based inhibition motif (ITIM) associated Siglec receptors presented on the same cell membranes and orchestrate inflammatory reactions within damaged tissues. Intriguingly, the pathogen-associated molecular patterns (PAMPs) developed the ability to recognize both, ITIM and ITAM (immunoreceptor tyrosine-based activation motif) associated Siglecs that underlie the mechanisms of chronic inflammation and neurodegeneration, as well as impaired immune surveillance in pathogen infections and cancer invasion [19].

### 3.1. CNS Diseases

Despite the role of the sialic acid-Siglec checkpoint, it was not widely studied in brain functions. There is increasing evidence that chronic stress exerts proinflammatory effects, which are associated with the local activation of microglia, the production of proinflammatory factors, neuronal atrophy, and increased expression of sialylated acute-phase proteins. The measurement of sialidase activity as a posttranslational indicator of glycoproteins remodeling revealed the pivotal role of PSA-NCAMs in chronic stress-induced cognitive disturbances [58]. Changes in PSA-NCAM expression in response to stress stimuli may reflect hippocampus atrophy in long-term exposure to corticosteroids known as endocrine modulators in stress [59]. On the cellular level, the polysialylated NCAMs are recruited in neuron-microglia interaction via Siglec-11 binding and ITIM-coupled signaling, and they restrict damage by immune cells during brain inflammation [60,61]. This scenario could reflect immune controlling mechanisms in the brain after exposure to proinflammatory factors. In an animal model of systemic inflammation, intraperitoneal injection of lipopolysaccharide (LPS) caused significant changes in the sialylation pattern in CNS. The elevated PSA-NCAMs expression in the hippocampus was correlated with the intracellular level of inflammation mediators [62]. Besides the role of PSA-NCAMs as regulators of neural plasticity in the hippocampus, their engagement in compensatory and protective mechanisms during neurodegeneration was also described. The presence of glycans, including α2.8-linked sialic acids, protects glycoconjugates against proteolysis and affects proper regeneration through the reestablishment of a crude topographic map of reinnervation [63]. The LPS-induced acute inflammation was not accompanied by the altered expression of suppressive Siglec-F both in vivo and in vitro studies and can be interpreted by the altered regulation of Siglec receptor expression in respective stages of inflammation [62]. Siglec receptors that contain ITAM promote proinflammatory cellular activity in the acute phase, whereas receptors with the ITIM inhibitory domain (e.g., Siglec-F, -G), reduce the cytotoxicity of immune cells in the chronic phase of inflammation [64].

The cell-intrinsic mechanism involving Siglecs can be associated with divergent outcomes of pathology within the brain. Moreover, the CD33-mediated suppression of microglia seems to be regulated alternatively by the hypersialylation of proteins and lipids. The sialic acid-rich glycoconjugates on the surface of amyloid plaques, mimicking the cell surface glycocalyx, activate the Siglec-11 receptor, and thereby switch the „Off” signaling which allows pathological structures to avoid immune surveillance of microglia [65]. It has been shown that Siglec-3 (CD33) and CD33-related Siglecs, including Siglec-11, belong to the top-rated factors which may confer the risk for Alzheimer’s disease (AD) [66,67]. Given the microglia ability for amyloid-β (Aβ) clearance, it seems that CD33-coupled signaling pathways can regulate their phagocytic potential. The postmortem analysis of the AD cortex evidenced an increased number of CD33-positive microglia, which was concomitantly linked with elevated CD33 mRNA level [68]. Nevertheless, the knocking out CD33 in the experimental mouse model of AD caused efficient phagocytosis of pathogenic Aβ by microglia and macrophages [69]. Therefore, the interactions between the sialoglycans and Siglecs are a promising targeting therapy based on antibodies with a monovalent affinity to different Siglecs.

### 3.2. Respiratory System Disorders

Clinical studies and animal models of respiratory tract obturation demonstrated that increased expression and specific distribution of Siglec-8 is closely associated with inflammation [70,71,72]. Progressive inflammation in airway tissues promotes the expression of specific sialoglycans carrying predominantly 6-sulfo-sialyl Lewis^x^ epitopes. The cross-linking with Siglec-8 initiates ITIM-signaling cascades and downstream effector proteins that lead to the apoptosis of infiltrating eosinophils. This process occurs when eosinophils are in proinflammatory cytokine milieu, indicating that Siglec-8 and its murine functionally convergent paralog, Siglec-F, regulate the turnover of activated cells in the context of inflammation. A growing body of evidence suggests that Siglec-8 is an important regulator of inflammation and disease. In animal models of the respiratory tract inflammation, mice lacking Siglec-8 have an increased inflammatory response and hypereosinophilic syndrome (HES) [73]. Contrary, the administration of Siglec-F antibodies in mouse models of chronic asthma normalizes eosinophilic pulmonary inflammation and eliminates lung tissue remodeling [74,75]. Interestingly, monocyte turnover in simian immunodeficiency virus (SIV) infections correlates with the severity of pulmonary lesions contribute to chronic pulmonary inflammation [76].

As with many human phenotypes, the controlling mechanisms of chronic inflammation, neurodegeneration, and immune surveillance depend on the levels of multiple genes. Siglec-5 and Siglec-14 belong to the group of paired receptors that show the extreme similarity of the amino acid sequence of the extracellular part and identical distribution in tissues and cells. This phenomenon results from partial conversion of the closely related SIGLEC5 and SIGLEC14 genes in the evolution process resulting in similar ligand recognition properties but an opposing signaling system [77]. According to the published data, the expression of the activating Siglec-14 receptor predominates in the European population and may potentiate inflammatory response in bacterial (*Haemophilus influenza*) and viral infections (influenza virus), which are the cause of chronic respiratory diseases. The clinical observation of patients with chronic obstructive pulmonary disease (COPD) has shown that the loss of Siglec-14 reduces the risk of COPD exacerbations related to bacterial infections [78]. The predominant expression of Siglec-5 is observed in the Asian population and is closely linked to reduced bactericidal and virucidal abilities during infections with Streptococcus, Neisseria, Pseudomonas, Campylobacter, and HIV [79]. It is of particular importance in exposure to proinflammatory conditions. The in vitro studies revealed an increase in the expression of paired Siglec-5/14 receptors in THP-1 cells exposed to cigarette smoke (CS). Simultaneous changes in immune activity as an increase in intracellular interleukin 1β (IL1β) and interleukin 10 (IL10) expressions and impairment of phagocytic capacity were observed. In parallel to CS-induced changes in human monocytes, an increase of sialoglycans in lung epithelial cells was observed [80]. It confirms the overwide hypothesis that CS cigarette smoke may induce functional alterations in the immune response in cells of the respiratory system. Changes in the expression of the paired Siglec-5/14 receptor may be important for predicting the risk of exacerbations in respiratory diseases and the immune system performance in bacterial and viral infections in both regular and social smokers.

### 3.3. Pathogen Invasion

In each immune disorder, the microbial invasion can contribute to the different stages of hyperinflammation. As suggested above, the interplay between pathogen sialoglycans and the host Siglec-5/14 can act as a regulatory mechanism of bacterial infections in the respiratory system. Besides the divergent role of paired Siglec-5/14 in the pathogen-dependent course of COPD, there is evidence of engagement of Siglec-5/14 in life-threatening organ dysfunction during infections with group *B Streptococcus* (GBS). Carlin et al. demonstrated that β-protein in GBS plays a pivotal role in the mechanism of molecular mimicry through the interaction with inhibitory Siglec-5 resulting in the impaired phagocytic function of lymphocytes [81]. In GBS-infected Siglec-5/14^+/+^ individuals, Siglec-14 on neutrophils counteract Siglec-5-mediated immunosuppression by activating p38 mitogen-activated protein kinase (MAPK) and Akt signaling pathways [82]. In sepsis, recently defined as endotoxin tolerance, monocytes undergo reprogramming to generate immunosuppression in the late phase of the disease. It has been shown that α2,3- and α2,6-sialylation on the LPS-induced tolerant RAW264.7 cell surfaces were significantly increased and correlated with enhanced Siglec-1 mRNA expression [83]. The interaction between Siglec-1 and the heavily sialylated proteins, e.g., mannose receptor, macrophage galactose-type lectin 1 (MGL1), mucin-1 (MUC1), and P-selectin glycoprotein ligand-1 (PSGL-1) enhances TGF-β1 production, and thereby, controls the development of endotoxin tolerance [84,85,86]. Clinical outcomes in sepsis confirm the association between high mortality and apoptosis—induced loss of cells of the innate and adaptive immune system, including CD4, CD8 T, and dendritic cells. Kidder et al. demonstrated that Siglec-1 positive macrophages induce the apoptosis of CD4^+^ T regulatory cells (Tregs) via recognition and binding of α2.3-linked sialic acids. However, the mechanism is not fully understood. In consequence, the reduction of Tregs numbers provides an increase in the T effectory cells (Teffs) population and promotes uncontrolled inflammation [87]. Siglec-2, which is mostly expressed on B cells, participates in the immune balance of sepsis through controlling chemokine production and regulating B cell response. Similarly, Siglec-10 plays an anti-inflammatory role in sepsis through increasing IL-10 expression. It has been shown that the anti-inflammatory effects in *Campylobacter jejuni* infections are mediated through the cis interaction between Siglec-10 and CD24 that inhibits dendritic cell cross-presentation and weak B cell signaling [88].

The sialic acid-Siglec axis has also been considered as a controlling mechanism in viral invasion machinery. Several sialylated glycoconjugates act as a key and facilitate the entry of retroviruses, including HIV, into the mature dendritic cell after binding to Siglec-1. In detail, the sialylated glycoprotein 120 (gp120) widely expressed on an HIV envelope can bind Siglec-1 and Siglec-7 on monocytes/macrophages and NK cells, respectively, which induces viral entry, promotes HIV replication and allows the infection of CD4^+^ T lymphocytes [89]. In the context of coronavirus (CoVs) pandemic issues, Varki and Angata hypothesize that the expression of sialic acids by the envelope of CoV can affect Siglec receptors biology in the hosts and thereby regulate the reactivity of innate immune cells [90]. The inhibitory receptors, such as Siglec-7 and -9, are also exploited in molecular mimicry mechanisms that allow viruses to avoid immune surveillance [91]. It has been demonstrated that the Hepatitis B virus (HBV) induces NK cell dysfunction via Siglec-9 recruitment. Conversely, blocking Siglec-9 on these cells of HBV-infected individuals increases TNF-α and IFN-γ secretion [92]. Thus, targeted manipulation of these processes could lead to a new therapeutic opportunity for patients with bacterial and viral infections [93,94].

### 3.4. Cancer Progression

Since sialic acids are commonly found in different types of cancers, their interplay with Siglec-expressing immune cells within the tumor microenvironment is considered a mechanism that shapes immune response in malignancy. The sialylation pattern in some cancers is highly heterogenous in specific cancer types and determines the profiles of engaged Siglec-expressing subpopulations of immune cells. Mostly, the Siglec expressing cells with the capacity for inhibitory signal transduction are recruited in cancer progression. It has been shown that lung cancers and melanomas express sialoglycans predominantly for Siglec-7 and Siglec-9. Among the human ligands, the highly sialylated mucin-1 (MUC-1), which binds Siglec-9, attenuates anti-tumor immunity in tumor-associated macrophages (TAMs) [95]. Moreover, the strong affinity of α2.3- and α2.6-linked sialic acids to Siglec-9 on neutrophils results in neutrophils inhibition measured by reactive oxygen species (ROS) production. In contrast, the administration of Siglec-9 targeting antibody restored the effector functions of these cells in the presence of malignant cells in vitro [96]. In macrophages, binding of cancer-associated MUC-1 to Siglec-9 induced the conversion into the M2 phenotype, which has the function of reducing inflammation and contributing to tumor growth and immunosuppressive function. Beside Siglec-9, macrophages express widely Siglec-5/14, Siglec-7, and Siglec-10 that give a wide sialoglycan binding spectrum and thereby increases the role of the sialic acid-Siglec axis in the anti-tumorigenic regulatory mechanism [41,46,97]. In the cellular model of glioma, the crosstalk between murine malignant astroglia and immune cells via sialic acid—Siglec-F or Siglec-E axis support tumor-promoting functions, including remodeling of the extracellular matrix and recruitment of immunosuppressive myeloid cells [98,99]. According to Engblom et al. observations, the presence of Siglec-F—positive neutrophilia within tumors promotes cancer growth and correlates with poor prognosis [100]. Interestingly, the enhanced expression of polysialylated neural cell adhesion molecules (PSA-NCAMs) in human glioblastoma promotes migration, invasion, and metastasis, and thereby has been described as an adverse prognosis factor [101]. Given the recognizing capacity of microglial Siglec-11, it is reasonable to speculate that the PSA-NCAM-Siglec-11 axis may underlie the immunosuppression and impaired immune surveillance in the brain. The participation of the Siglec-sialoglycans axis in the maintenance of immune homeostasis suggests that the targeted manipulation of these processes could open a new therapeutic way in multiple immune-based disorders. Numerous clinical trials for cancer and autoimmune disorders revealed the beneficial effects of anti-CD22 (Siglec-2) and CD33 monoclonal antibodies (mAb), in particular when conjugated with immunotoxins. However, multiple adverse effects, including increased mortality, were observed [102]. Recently, Siglec-9 and Siglec-15 have been reported as crucial inhibitors of anti-tumor immunity, which can be blocked by mAbs in the novel anticancer management [103,104]. Recent advances in the field of immunotherapy suggest that targeting Siglec receptors with specific antibodies or fluorinated sialic acid analogs, called “false sialic acids,” help to control autoimmunity, pathogen invasion, and malignancies [105,106].

### 3.5. Cardiovascular System Dysfunction

Moreover, a growing body of evidence supports the role of the sialoglycan-Siglec axis in the pathogenesis of vascular dysfunctions. The epidemiological analysis has uncovered a positive correlation between plasma total sialic acid and the risk of coronary artery disease (CAD) [107]. It has been shown that murine Siglec-G, mainly expressed on B-1 cells, promotes atherosclerosis and liver inflammation by inhibiting the protective function of B-1 cells [108]. The clinical studies showed that CAD patients express a reduced Treg level and Treg/Teff ratio, caused by the modulatory function of Siglec-E-expressing dendritic cells, as confirmed in animal models of CAD. Inhibition of Siglec-1, highly expressed on circulating monocytes and plaque macrophages in atherosclerotic patients, can prevent atherosclerotic lesion formation by suppressing the interaction between monocytes and endothelial cells, and macrophages accumulation [109,110]. In a laboratory model of diabetes, hyperglycemia-induced up-regulation of sialoglycans on human umbilical vein endothelial cells (HUV-EC-C) and mouse aorta was associated with the decreasing of Siglec-9-mediated phagocytic activity in macrophages and was described as a significant risk factor of angiopathy [111].

## 4. Sialic Acid-Siglec Checkpoint and Conventional Therapy

Despite the better knowledge of molecular mechanisms of immunity and progress in the development of new targeting drugs, conventional therapies are still the main strategy in the management of multiple disorders. In addition to well-determined and desired clinical outcomes, standard therapies include multiple negatives ranging from expected and/or unexpected adverse effects to not fully understood and studied interference on treatment efficacy. There are minimal data on the effects of conventional drugs on the sialoglycan-Siglec checkpoint and its importance in the progression of the disease process (Table 1).

### 4.1. Sialidase Inhibitors—Not Only in Influenza Virus Infections

The inhibition of glycan–lectin interactions is of importance in the treatment of pathogenic infections and several other glycan based diseases. The disruption of glycome controlling mechanisms prevents the interaction between pathology-related molecules. Oseltamivir and zanamivir, the most active inhibitors of influenza sialidase, prevent the virus release from the host cells and its multiplication [112]. In addition to the direct effect on viral sialidase, oseltamivir modulates DCs activity via sialidase-mediated Siglec-Toll like receptor (TLR) interaction [113,114]. Several Siglec receptors, e.g., murine Siglec-E and human Siglec-5/-9, interact with toll-like receptors (TLR) and inhibits their activation, thereby helping to maintain a healthy cytokine balance following infection. In the presence of pathogens, endogenous neuraminidase-1 (sialidase-1, Neu-1) disrupts the interaction between the TLRs and the Siglecs, thereby activating the receptors and triggering an immune response during infection [115,116]. However, abnormal TLR4 activation by bacterial endotoxin in sepsis can be reduced by oseltamivir-induced Neu-1 inhibition and protects against endotoxemia [83,117]. Additionally, recent clinical investigations revealed that targeting sialidase-1 (neuraminidase-1, Neu-1) by reducing total sialic acid contents may represent a possible therapeutic strategy in CAD therapy [118]. In cancer, Neu-1 inhibition by oseltamivir changes epidermal growth factor receptor (EGFR)-mediated signaling and shift cadherin expression that reduces metastatic potential and chemoresistance in various malignant cells [119,120]. There are no data about the recruitment of sialidase inhibitors in sialic acid-Siglec checkpoint activity.

### 4.2. Sialic Acid-Siglec Axis and Standard Respiratory Obstruction Therapy

**Figure 1:**
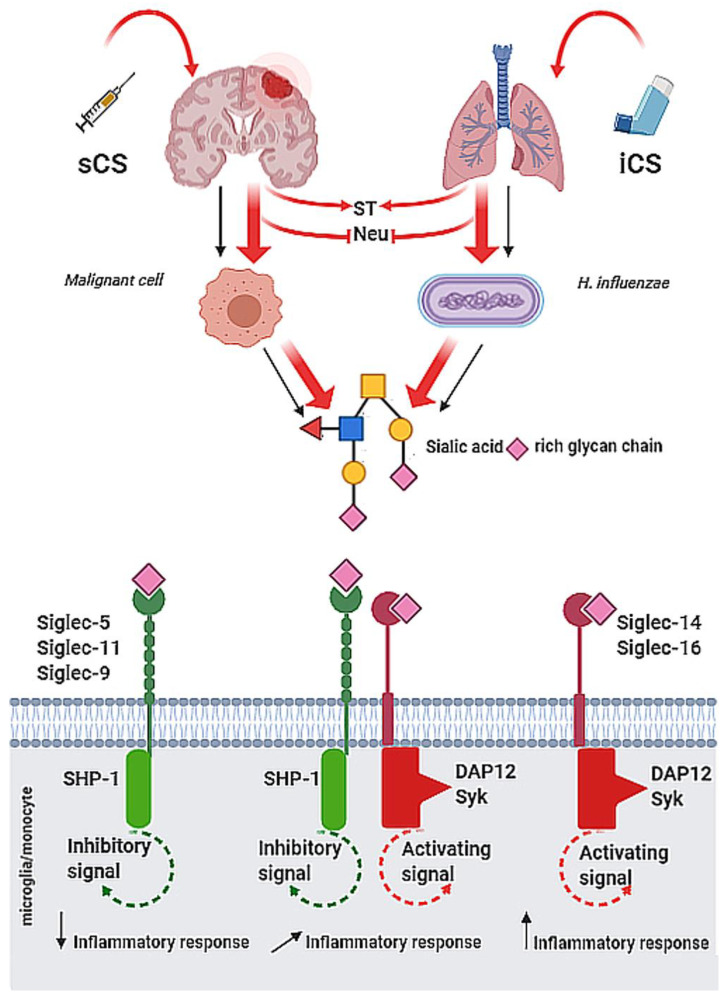
Possible effects of systemic (sCS) and inhaled corticosteroids (iCS) on immune functions via sialoglycan-Siglec checkpoint. The administration of sCS and/or iCS modulates the expression and activity of sialyltransferases (ST) and neuroaminidases (Neu) that result in changes of cell surface sialylation pattern. Depending on inhibitory and activatory Siglec expression in individuals, the binding of specific sialic acid ligands exerts the immune response of different intensity in selected pathological stages.

Siglecs are involved in respiratory tract disorders. The molecular mechanisms of glycome machinery and its therapeutic targeting are extensively studied. According to the GINA (Global Initiative for Asthma) and GOLD (Global Initiative for Chronic Obstructive Lung Disease) guidelines, the main goal of conventional therapies in respiratory obstructive diseases is the limiting of inflammation [121,122,123]. However, in contrast to bronchial asthma, the inefficient management of COPD is related to the low sensitivity of patients to corticosteroids, and relatively high risk of exacerbation due to bacterial or viral infections [124]. The effects of mono- and combined therapies on paired Siglec-5/14 receptors were evaluated in CD14^+^ cells isolated from clinically stable COPD patients. It has been shown that inhaled corticosteroids (ICS), but not long-acting β2-agonist (LABA) and long-acting muscarinic antagonists (LAMA), increase Siglec-5 and/or Siglec-14 expression. Given the function of paired receptors, ICS, depending on the patients’ genotype, may exert either beneficial or negative effects through the enhanced expression of paired Siglec-5/14 receptors and may raise the risk of harm to some individuals [125]. Zeng et al. demonstrated that dexamethasone (Dex), a potent routinely used corticosteroid, might exert an anti-inflammatory effect on COPD-origin neutrophils by up-regulating Siglec-9 expression (Figure 1) [126].

Moreover, a high level of Siglec-8 was observed in cells isolated from induced sputum in eosinophilic COPD patients after add-on LAMA therapy, which may have a pivotal role in disease regulation by downregulation of eosinophils [64]. The Siglec-8-related eosinophils maturation was also detected in the aspirin-exacerbated respiratory disease (AERD) but not in eosinophilic aspirin-tolerant asthma and chronic sinusitis [127].

### 4.3. Corticosteroids—Benefits and Pitfalls in the Cancer Management

In recent years, the engagement of Siglecs in cancer progression was intensively studied. Some of them have been described as diagnostic markers and a promising therapeutic target. Current clinical trials based on the targeting of sialic acid-Siglec axis revealed that ligation of sialylated ligands to ITIM-coupled Siglecs on leukocytes mediates immunosuppression and blockade of anti-tumor activity, whereas targeting of Siglec-3/-7/-9 or -15 by MAbs promotes anti-tumor immunity [96]. The clinical trials and preclinical observations running for the treatment of cancer showed that corticosteroids interfere with the function of local and infiltrating immune cells and impair cancer immunosurveillance [128,129,130,131]. According to neurosurgery and brain oncology guidelines for pre- and postoperative management, systemic corticosteroids are a “gold standard” in the regular therapy of glial tumors [132]. The retrospective clinical studies confirm the beneficial antioedemic effects of Dex. However, they also suggest the activation of mechanisms that activate genes expression correlated with shorter survival [129,133,134,135,136,137]. The mechanisms of therapeutic effects of corticosteroids and modulatory action on cell biology are well established, but non-genomic mechanisms underlying cancer immune evasion are not fully understood. Since sialic acid is involved in the regulation of immunogenicity effect of corticosteroids on sialoglycans in gliomas was studied [138,139]. Cytometric analysis of glioblastoma cells of different immunogenicity showed a dose-dependent effect of dexamethasone on the sialylation pattern, which was also associated with the changed affinity of the Siglec-E and -F receptors to glioma cell membranes [98]. In co-culture systems without physical interaction, dexamethasone enhanced α2.8-sialylation in glioma cells, which was accompanied by the promotion of the suppressive immune status of microglial cells [99]. It may reflect Dex-induced dampening of anti-tumor immunity via interferention with the activity of the sialoglycan-Siglec checkpoint and mechanisms controlling the glycome machinery. According to the Cancer Genome Atlas, Dex activates several genes, including CDC25C, CDCA8, CDC20, PRC1, and PLK1 that are closely correlated with a worse prognosis and shorter survival in patients with glioblastoma [133]. Given the effects of corticosteroids on glycosylation pattern and sialome-dependent cellular interactions, the assessment of individual Siglec profiles in patients with malignant gliomas may be useful in verifying the safety of steroid therapy and the prediction of overall survival.

Given that the genomic and nongenomic mechanisms of action of corticosteroids are not fully understood, their clinical importance in the management strategies of lymphoid neoplasms is relatively high. Since the correlation of CD33 with poorer prognosis in leukemia was established, the running clinical trials implicate the potential of anti-CD33 frontline therapy [102,140]. However, it has been also shown that various dosage systems of corticosteroids, including prednisone and methylprednisolone, exert a pro-apoptotic effect toward CD33-positive lymphoblasts [141]. In B-acute lymphoblastic leukemia, phase II of the clinical trial on targeting for Siglec-2 revealed that the combined therapy with Dex increases the therapeutic efficacy of epratuzumab in Siglec-2-positive B cells [142].

### 4.4. Anti-Inflammatory Management

The extensive studies on the pathogenesis of AD revealed the beneficial role of anti-inflammatory, analgesic, and local anesthetic medications in the prevention of degenerative processes within the CNS. Using the preclinical mice model of surgery-induced neuroinflammation, Xu et al. showed that post-operative cognitive dysfunction in old, but not young, animals are strongly correlated with the increased levels of TNFα, IL-6, Iba-1, and CD33-positive cells in the hippocampus [143]. Despite many limitation of this study, the authors suggest that ibuprofen, an anti-inflammatory and analgesic drug, as well as local anesthetic levobupivacaine suppress inflammation and microglia activation but do not affect the cognitive function in experimental animals. As mentioned previously, Siglec-3 and other CD33-related Siglecs are associated with AD pathology. Therefore, it may be beneficial to consider anti-inflammatory therapy to limit the risk of post-operative cognitive dysfunction in elderly individuals. This observation opens a new view of the standard pharmacological strategies, as well as searching for biologically active natural substances that exert neuroprotective effects through the recruitment of some immune checkpoints. In line with this, curcumin, the widely known component extracted from the rhizome of *Curcuma longa*, has been described as a candidate for the diagnosis, prevention, and treatment of AD. Besides the antioxidation and anti-inflammatory properties, the strong biological activity of curcumin expressed by downregulation of Siglec-3 capacity results in the phagocytic clearance of amyloid and makes it a potential. However, it is still not formally registered as a therapeutic tool for AD as confirmed in human sections ex vivo [144].

Recent studies on the risk factors for fetal development confirmed the role for type 1 interferone (IFN) in the pathogenesis of autoimmune congenital heart block (CHB) in newborns [145]. The analysis of immune cells subpopulations from a fetus with CHB showed that upregulation of type 1 interferone correlates with a high level of Siglec-1 expressing monocytes and/or macrophages that are functionally involved as effector cells in fibrosis. The clinical investigations on the CHB preventing strategies revealed that targeting the maternal interferone significantly reduces the risk of fetal affections. As Lisney et al. have reported, the IFN-α-targeted therapy with anti-inflammatory hydroxychloroquine decreases Siglec-1 expression on maternal monocytes and/or macrophages and reduces risk for the development of fetal CHB [146].

The function of the sialic acid-Siglec axis in the host response to the conventional drugs-related side effects was analyzed in the pharmacologically induced tissue injury. Scaffidi et al. showed that high dose of acetaminophen, routinely used to treat mild to moderate pain or to reduce fever, causes the hepatocytes injury accompanied by the release of high-mobility group box 1 protein (HMGB-1) [147]. During cellular injury, HMGB-1, similar to the heat shock protein 70 and 90 (HSP-70, -90) are capable of induce inflammatory response expressed production of IL-6 and TNFα. However, the heavy sialylated CD24-Siglec-10 axis on human macrophagesas, as well as its murine analogue CD24-Siglec-G, shows HMGB-1 binding capacity, thereby dampen tissue damage-induced immune responses. Contrarily, mice with a targeted mutation of Siglec-G encoding gene and CD24 deficiency are extremely sensitive to acetaminophen-induced liver injury and are predisposed to develop cytokine release syndrome [148] (Table 1.).

## 5. Conclusions and Perspectives

This brief review focuses on some examples of the potential role of the sialic acid-Siglec checkpoint in pathological states and related conventional therapies. The interplay between sialoglycans and Siglecs undergoes dynamic changes in many physiological and pathological processes. Both, in resting and activation status, the glycome machinery controls the sialylation pattern and Siglec-related cellular activity that underlies the immune homeostasis and participates in the immune defense. Thus, it is certainly an important target in the field of glycoengineering-based therapy. The mechanisms of a new targeting therapies inhibit the Siglec-mediated cellular processes by structurally modified sialoglycans and monoclonal anti-Siglec antibodies applied in the modern delivery systems, as well as enzymatic modifications of cell membranes, seem to be showing therapeutic potential in future medicine. However, conventional therapy will be the main strategy in clinical management, and its interference with components of sialic acid-Siglec immune checkpoints should be verified in cancer or inflammatory diseases.

## Figures and Tables

**Table 1:** Human/murine Siglec receptors in selective pathologies and related conventional therapies.

Pathology	Conventional Therapy/Drugs	Human/Murine Siglec Involved	References
Infections	zanamivir, oseltamivir	Siglec-5,-9,-E	[115,116]
COPD	corticosteroids, LAMA, LABA	Siglec-5/14, Siglec-8	[64,125,126]
Asthma	aspirin	Siglec-8	[127]
Brain tumors (in vitro models)	corticosteroids	Siglec-E,-F	[98,99]
Leukemia	corticosteroids	CD22, CD33	[141,142]
Alzheimer’s’ Disease	ibuprofen, levobupivacaine	CD33	[143]
Congenital Heart Block	Hydroxychloroquine	Siglec-1	[146]
Liver injury	acetaminophen	Siglec-10	[147,148]
